# Deciphering the Role of CBF/DREB Transcription Factors and Dehydrins in Maintaining the Quality of Table Grapes cv. Autumn Royal Treated with High CO_2_ Levels and Stored at 0°C

**DOI:** 10.3389/fpls.2017.01591

**Published:** 2017-09-20

**Authors:** Maria Vazquez-Hernandez, Irene Romero, M. I. Escribano, Carmen Merodio, M. T. Sanchez-Ballesta

**Affiliations:** Departamento de Caracterización, Calidad y Seguridad, Instituto de Ciencia y Tecnología de Alimentos y Nutrición, ICTAN-CSIC, Ciudad Universitaria de Madrid Madrid, Spain

**Keywords:** CBF/DREB, dehydrins, transcription factors, *Vitis vinifera*, carbon dioxide, low temperature

## Abstract

C-repeat/dehydration-responsive element binding factors (CBF/DREB) are transcription factors which play a role in improving plant cold stress resistance and recognize the DRE/CRT element in the promoter of a set of cold regulated genes. Dehydrins (DHNs) are proteins that accumulate in plants in response to cold stress, which present, in some cases, CBF/DREB recognition sequences in their promoters and are activated by members of this transcription factor family. The application of a 3-day gaseous treatment with 20 kPa CO_2_ at 0°C to table grapes cv. Autumn Royal maintained the quality of the bunches during postharvest storage at 0°C, reducing weight loss and rachis browning. In order to determine the role of CBF/DREB genes in the beneficial effect of the gaseous treatment by regulating DHNs, we have analyzed the gene expression pattern of three *VviDREBA1s* (*VviDREBA1-1*, *VviDREBA1-6*, and *VviDREBA1-7*) as well as three *VviDHNs* (*VviDHN1a*, *VviDHN2*, and *VviDHN4*), in both alternative splicing forms. Results showed that the differences in *VviDREBA1s* expression were tissue and atmosphere composition dependent, although the application of high levels of CO_2_ caused a greater increase of *VviDREBA1-1* in the skin, *VviDREBA1*-*6* in the pulp and *VviDREBA1*-*7* in the skin and pulp. Likewise, the application of high levels of CO_2_ regulated the retention of introns in the transcripts of the dehydrins studied in the different tissues analyzed. The DHNs promoter analysis showed that *VviDHN2* presented the *cis*-acting DRE and CRT elements, whereas *VviDHN1a* presented only the DRE motif. Our electrophoretic mobility shift assays (EMSA) showed that VviDREBA1-1 was the only transcription factor that had *in vitro* binding capacity to the CRT element of the *VviDHN2* promoter region, indicating that the transcriptional regulation of *VviDHN1a* and *VviDHN4* would be carried out by activating other independent routes of these transcription factors. Our results suggest that the application of high CO_2_ levels to maintain table grape quality during storage at 0°C, leads to an activation of *CBF/DREBs* transcription factors. Among these factors, VviDREBA1-1 seems to participate in the transcriptional activation of *VviDHN2* via CRT binding, with the unspliced form of this *DHN* being activated by high CO_2_ levels in all the tissues analyzed.

## Introduction

Storage temperature is one of the most important factors which affects postharvest fruit quality. Accordingly, low temperature is the most common abiotic stress applied to improve fruit quality during postharvest storage. To cope with low temperature, fruit have developed adaptive mechanisms, involving physiological and biochemical processes together with transcriptomic modifications, including regulatory and functional genes (Maul et al., 2008; Genero et al., 2016; Rosales et al., 2016). Among the regulatory genes, transcription factors play an important role in plant stress responses, acting as coordinators of stress signals and orchestrating the expression of functional genes (Singh et al., 2002; Wang et al., 2016). It is well known that C-repeat/dehydration-responsive element binding factors (CBF/DREB) are transcription factors which are involved in improving cold stress resistance (Stockinger et al., 1997; reviewed by Chew and Halliday, 2011). CBF/DREB proteins, which belong to the subgroup A1 of the DREB proteins and are members of the AP2/ERF (APETALA2/Ethylene-Responsive Factor) transcription factor superfamily, are able to recognize and bind to the DRE/CRT (Dehydration Responsive Element/C-Repeat) DNA regulatory motif in the promoters of many cold-responsive (*COR*) genes (Yamaguchi-Shinozaki and Shinozaki, 1994; Stockinger et al., 1997). In this respect, dehydrins (DHNs), a subgroup of LEA (Late Embryogenesis Abundant) proteins, are among the most commonly observed proteins which accumulate in plants in response to low temperature and environmental factors leading to cellular dehydration. The presence of DRE/CRT motifs in the promoter of several cold-regulated *DHNs* suggests that they play a role in the CBF/DREB-mediated signaling pathway (Gilmour et al., 2004; Wisniewski et al., 2006).

*CBF/DREB* transcription factors were first isolated from Arabidopsis (Stockinger et al., 1997; Medina et al., 1999; Haake et al., 2002) and thereafter they have been isolated from different woody species including almond (Barros et al., 2012), dwarf apple (Yang et al., 2011), sweet cherry (Kitashiba et al., 2004), and trifoliate orange (He et al., 2012). The overexpression of *CBF/DREBs* increased the tolerance to cold stress in Arabidopsis (Novillo et al., 2007), potato (Pino et al., 2008), and bilberry (Oakenfull et al., 2013). Thus, *CBF/DREBs* form part of the group of transcription factors identified as the first wave of cold-induced genes (Chinnusamy et al., 2007; Thomashow, 2010; Zhao and Zhu, 2016). The constitutive overexpression of *CBFs* in Arabidopsis activated the expression of DRE/CRT-containing target genes (including those that encode DHNs proteins such as *COR47* and *ERD10*, or LEA proteins like *COR15a*) under normal growing conditions and enhanced freezing tolerance in the absence of a cold stimulus (Jaglo-Ottosen et al., 1998; Gilmour et al., 2004).

There is no doubt that *CBF/DREB* transcription factors are vital for plants to overcome cold stress, but little is known regarding how important they are for fruit. Storage of peach fruit at a chilling injury-delaying temperature (0°C) resulted in a greater accumulation of *PpCBF1*/*5*/*6* transcripts than in fruit treated with a chilling injury-inducing temperature (5°C) (Liang et al., 2013). Zhang et al. (2017) indicated that several postharvest treatments, including ethylene (Zhao et al., 2009) and nitric oxide (Zhao et al., 2011), as well as hot water (Ma et al., 2014), triggered *CBF* expression in tomato fruit in the two first cases, and in kiwifruit in the latter, which enhanced cold tolerance during low temperature storage.

Grape (*Vitis* sp.) is one of the most economically important fruit crops in the world. Most of the studies on *Vitis*, which have deciphered the effect of *CBFs* on cold tolerance have been carried out with grapevine seedlings, and there is little information about their role in bunches. Thus, an increase in *CBF3* and *CBF4* expression was observed in the leaves of *Vitis riparia* and *V. vinifera* after 1–2 days at 4°C (Xiao et al., 2006, 2008), contrasting with the quick cold-induction observed in the case of *CBF1* and *CBF2* (Xiao et al., 2006). Karimi et al. (2015) compared the response of plants of one cultivar of *V*. *riparia* and two cultivars of *V. vinifera* to cold stress, and denoted that *V*. *riparia* which is endemic to cold regions, behaved stronger after 10 days of exposure to 4°C and showed higher expression for all the *CBFs* analyzed. Overexpression of *VvCBF4* in *V. vinifera* cv. Freedom improved freezing survival and reduced freezing-induced electrolyte leakage by up to 2°C in non-cold-acclimated vines (Tillett et al., 2012). Likewise, overexpression of *CBF* transcription factors from *V. vinifera* cv. Koshu (Takuhara et al., 2011) or *V. riparia* (Siddiqua and Nassuth, 2011) in Arabidopsis improved the freezing tolerance of transgenic plants. Furthermore, the results from Siddiqua and Nassuth (2011) indicated that the Arabidopsis lines that overexpressed *VrCBF1* or *VrCBF4* showed an increase in the accumulation of *COR* genes, including dehydrin (*COR47*) and *LEA_4* (*COR15a*), which have at least one DRE/CRT motif in their promoter (Kasuga et al., 1999).

The application of a 3-day gaseous treatment with 20 kPa CO_2_ at 0°C maintained the quality of red table grapes cv. Cardinal during postharvest storage at 0°C by reducing total decay and rachis browning (Sanchez-Ballesta et al., 2006; Rosales et al., 2016). Although table grapes are classified as chilling-tolerant, we have pointed out that the gaseous treatment help table grapes to face temperature shifts at 0°C (Sanchez-Ballesta et al., 2006) and thus CO_2_-treated berries reached a low ion leakage value during storage at 0°C in comparison to non-treated ones (Rosales et al., 2016). In a previous study, we observed that application of high CO_2_ levels at 0°C in table grapes cv. Cardinal for 3 days induced the expression of *CBF1* and *CBF4* (*VviDREBA1-1* in this work) in the pulp and *CBF4* in the rachis (Fernandez-Caballero et al., 2012). In a more recent work, we suggested that the beneficial effect of a high CO_2_ treatment to maintain Cardinal table grape quality seems to be mediated by the regulation of *ERFs* (Romero et al., 2016), which belong to the AP2/ERF transcription factor family. In the present study, we have analyzed the effect of a 3-day CO_2_ treatment in maintaining the table grape quality of a cultivar which is different from Cardinal, such as Autumn Royal. Unlike red-skinned Cardinal table grape cultivar which matures early (late spring to mid-summer) and presents very few seeds, Autumn Royal is a late-maturing seedless table grape cultivar (from autumn through early winter), with a purple-black to black berry skin. Furthermore, to investigate whether the role played by *CBF/DREB* transcription factors is cultivar dependent and/or a common feature of other *DREBA1s*, we have analyzed the transcript accumulation pattern of *VviDREBA1-1*, *VviDREBA1-6*, and *VviDREBA1-7* in different tissues (skin, pulp, and rachis) of Autumn Royal bunches which were treated and non-treated with 20 kPa CO_2_ for 3 days at 0°C and then transferred to air for up to 13 days. Likewise, to study the role of CBF/DREBs in the regulation of *DHNs* in table grapes we first analyzed the pattern of expression of three dehydrins (*VviDHN1a*, *VviDHN2*, and *VviDHN4*) in different tissues of Autumn Royal bunches which were CO_2_-treated and non-treated; and we then analyzed their promoters so as to identify different *cis*-regulatory elements, including the DRE/CRT motifs. Finally, we examined the DNA-binding specificities of three CBF/DREBs, by using electrophoretic mobility shift assay (EMSA), showing that only VviDREBA1-1 was able to bind *in vitro* to the CRT element present in the *VviDHN2* promoter.

## Materials and Methods

### Plant Material

Mature table grapes (*V. vinifera* L. cv. Autumn Royal) (12.87% total soluble solids; 0.46% tartaric acid) were harvested from a commercial orchard located in Abarán, Murcia, Spain (latitude: 38° 12′ 00′′ N; longitude: 01° 24′ 00′′ W) in November 2013. After random harvesting, field-packaged bunches were transported in the same day to the laboratory in Madrid (Spain), where bunches without physical and pathological defects were divided arbitrarily into two lots and stored at 0 ± 0.5°C with 95% relative humidity in two sealed methacrylate containers of 1 m^3^ capacity. One lot was stored under normal atmosphere for up to 41 days (non-treated fruit) and the second one was kept under a gas mixture containing 20 kPa CO_2_ + 20 kPa O_2_ + 60 kPa N_2_ (CO_2_-treated fruit) for 3 days. Thereafter, CO_2_-treated table bunches were transferred to air under the same conditions as non-treated ones until the end of the storage period. At time 0 and after 3 and 13 days of storage under air or CO_2_ conditions, skin, pulp and rachis from three biological replicates (each replicate consisting of 2 bunches) were collected independently, frozen in liquid nitrogen, grounded to a fine powder and stored at -80°C until analysis.

### Quality Assessments

Berry quality assessment comprised soluble solids contents (SSC), titratable acidity (TA), pH, weight loss of bunches and rachis browning. SSC was determined using a digital refractometer Atago PR-101 (Atago Co. Ltd., Japan) at 20°C and expressed in °Brix. TA was determined by titration with 0.1 N NaOH up to pH 8.1 and results were expressed in % tartaric acid. The pH of the juice was measured using a pH meter with a glass electrode. The moisture content of berries was determined when a stable weight had been obtained after drying the fruit at 105°C, and it was expressed as g/100 g fresh weight (FW). The weight of the bunches was recorded on the day of harvest and after the different sampling dates. Cumulative weight losses were expressed as a percentage loss of the original weight. Rachis browning was determined using the following subjective scale: (0) none (rachis including pedicels, green-bright), slight (1) (rachis in good conditions and pedicels, green–gray), (2) moderate (secondary rachis and pedicels, green–brown), (3) intense (secondary rachis and pedicels, brown, and primary rachis, green with brown areas), (4) severe (pedicels, primary and secondary rachis, brown).

### RNA Extraction, cDNA Synthesis and RT-PCR

For each sample, total RNA was extracted three times according to Zeng and Yang (2002), and treated with DNase I recombinant-RNase free (Roche) for genomic DNA removal. Concentration and purity of the total RNA samples were measured using the NanoDrop^TM^ 1000 Spectrophotometer (NanoDrop Technologies, Inc. Wilmington, DE, United States). Then, 1 μg of each extraction was used to synthesize cDNA by using the iScriptTM Reverse Transcription Supermix (Bio-Rad), according to the manufacturer’s instructions. *VviDREBA1s* full-length sequences were obtained by RT-PCR as described by Romero et al. (2008) using specific primers (Supplementary Table S1). Genomic DNA, obtained from leaves of *V. vinifera* cv. Autumn Royal as described by Lodhi et al. (1994) was used as template. PCR fragments were cloned and confirmed by sequencing as described by Romero et al. (2016).

### Bioinformatic Tools

The *V. vinifera CBF/DREBs* previously identified (Xiao et al., 2006, 2008; Zhuang et al., 2009; Licausi et al., 2010; Cramer et al., 2014; Zhao et al., 2014; Carlow et al., 2017) were used to run a search in the 12X grape reference genome, V2.1 gene prediction hosted at Grape Genome Database (CRIBI^1^) (Vitulo et al., 2014). The BLASTP and BLASTX suites were used to perform similarity searches in the predicted proteome database. Protein sequence identity between the closest *VviDREBA1s* homologs were performed by using the LALIGN program^2^.

Multiple alignment analysis was performed at the Swiss EMBnet node web server using ClustalW and BoxShade tools, respectively. The prediction of ubiquitination and sumoylation sites was performed using, UbPred^3^ (Radivojac et al., 2010) and SUMOsp2.0 servers^4^ (Zhao et al., 2014), respectively. The proline (P) glutamic acid (E) serine (S) threonine (T) (PEST) regions in the proteins were found using the EMBOSS program pestfind^5^ with the default cut-off PEST score of 5.0 (Rice et al., 2000). Hydrophobic cluster analysis (HCA) was performed using online tools at http://bioserv.rpbs.univ-paris-diderot.fr/services/HCA/ (Gaboriaud et al., 1987).

### Relative Gene Expression by Quantitative and Semi-Quantitative RT-PCR

Relative expression of *VviDREBA1s* as well as spliced and unspliced transcripts of *VviDHN4* were assayed using quantitative RT-PCR (RT-qPCR) with samples of skin, pulp, and rachis from CO_2_-treated and non-treated bunches stored for 0, 3, and 13 days at 0°C. RT-qPCR was performed as described by Rosales et al. (2013), using gene-specific primer pairs (Supplementary Table S1). *Actin1* gene from *V. vinifera* (ACT1: XM 002282480) was used as the internal reference gene for normalizing the transcript profiles following the 2^-ΔΔCt^ method relative to a calibrator sample (day 0, bunches before storage). The specificity of products was validated by dissociation curve analysis and by agarose gel; and its sequences confirmed at the Genomic Department of the CIB-CSIC (Madrid, Spain).

Spliced and unspliced variants of *VviDHN1a* and *VviDHN2* were evaluated by semi-quantitative RT-PCR as described by Navarro et al. (2015). Following amplification, products were visualized by electrophoresis in a 2% agarose gel stained with Goldview (Guangzhou Geneshun Biotech Ltd.). The identification of each PCR product was then confirmed by Sanger sequencing at the Genomic Department of the CIB-CSIC (Madrid, Spain).

### Identification of Putative *Cis*-Regulatory Elements

Identification of the potential *VviDHN2* and *VviDHN4* promoter regions and transcription factor binding sites was conducted using the Genomatix suite of programs^6^ (Genomatix Software GmbH, Munich, Germany). The Gene2promoter program from the Genomatix software package was used to define 1500 bp upstream of the transcription start site of *VviDHN2* and *VviDHN4* promoter regions. The corresponding sequences were then used as the target sequences for putative transcription factor recognition site identification using the MatInspector Version 8.3 program (Cartharius et al., 2005) with the standard (0.75) core similarity and the optimized matrix similarity. DRE regulatory element from *VviDHN1a* promoter was previously identified (Rosales et al., 2014).

### Production of the Recombinant *VviDREBA1s* Proteins in *Escherichia coli*

The full *VviDREBA1s* [GenBank Accession No. MF445007 (VviDREBA1-1), MF445008 (VviDREBA1-6) and MF445009 (VviDREBA1-7)] open reading frames (ORFs), including stop codons, were amplified by RT-PCR using the primers included in the Supplementary Table S1. The forward *VviDREBA1-7* and *VviDREBA1-1* primers contained a BamHI site, and the forward *VviDREBA1-6* primer contained a XhoI site. The three reverse primers contained an EcoRI site. The resulting fragments digested with their respective restriction enzymes were cloned into the pTrcHisA vector, which contains an N-terminal His_6_ (Invitrogen, Carlsbad, CA, United States), previously digested with the same enzymes, and transformed into BL21-CodonPlus (DE3)-RIL competent cells. The induction and purification of recombinant proteins were performed according to Romero et al. (2008). The purified fusion proteins were concentrated as described by Romero et al. (2016). Protein analyses were performed on 12.5% sodium dodecyl sulfate polyacrylamide gel electrophoresis (SDS–PAGE) using Mini-Protean II Cell (Bio-Rad) equipment as described by Rosales et al. (2014). Western blots were probed with antibodies and conditions previously described by Romero et al. (2016).

### Electrophoretic Mobility Gel Shift Assay (EMSA) Assays

Purified VviDREBA1s recombinant proteins were used to determine DNA binding by EMSA as previously described by Romero et al. (2016). DRE/CRT motifs were synthetized and used as probes, which were biotin-labeled using the Biotin 3′ End DNA Labeling Kit (Thermo Scientific Pierce).

### Statistical Analyses

The data were analyzed by ANOVA (one-way analysis of variance) and Duncan’s multiple range test was used (IBM Corp. SPSS Statistics version 22.0., Armonk, NY, United States). Statistical significance was assessed at the level *P* ≤ 0.05.

## Results

### Effect of a 3 Day-CO_2_ Treatment on the Quality of Table Grapes cv. Autumn Royal Stored at 0°C

The application of high CO_2_ levels for 3 days did not affect the SSC and TA values after 13 days of cold storage, in comparison to non-treated grapes (**Table 1**). Likewise, the maturity index (SSC/TA) in freshly harvested fruit (27.87) increased in both non-treated and CO_2_-treated grapes after 13 days of cold storage, due to the increase observed in the SSC content, reaching values of 29.63 and 29.06, respectively. By contrast, whereas the pH values did not change in non-treated fruit, a significant increase was observed in CO_2_-treated grapes, decreasing by day 13. Regarding the moisture content of berries, it decreased significantly during storage at 0°C in all the samples analyzed in comparison to freshly harvested fruit, although the major decrease was observed in non-treated samples after 13 days of cold storage. The CO_2_ treatment was effective in controlling the weight loss of bunches, as well as the rachis browning observed in non-treated table grapes. After 3 days, the percentage of weight loss and the rachis browning index were significantly lower in CO_2_-treated bunches, but the effect was only maintained after 13 days in the case of weight loss. By contrast, although the rachis browning index was also lower in CO_2_-treated bunches after 13 days at 0°C, the differences were not significant.

**Table 1:** Soluble solids content (SSC), titratable acidity (TA), pH, moisture content, weight loss and rachis browning index of table grapes cv. Autumn Royal treated with 20 kPa CO_2_ and stored during 13 days at 0°C.

		0°C				
	Freshly harvested	3 days Air	3 days CO _2_	13 days Air	3 days CO_2_ + 10 days Air
SSC (%)	12.87 a	12.94 a	13.09 ab	13.64 bc	13.78 c
TA (% tartaric acid)	0.46 a	0.48 a	0.48 a	0.46 a	0.47 a
Maturity index (SSC/TA)	27.87	27.11	27.09	29.63	29.06
pH	3.88 b	3.84 b	3.94 c	3.87 b	3.79 a
Moisture content (g/100 g FW)	89.11 c	88.56 ab	88.41 ab	88.19 a	88.40 ab
Weight loss (%)	0	1.60 b	1.00 a	2.30 c	1.50 b
Rachis browning index	0	0.90 b	0.50 a	1.30 b	1.10 b

*Values are the mean of three replicate samples ±SE. Different letters within each row indicate that means are statistically different using Duncan’s test (*P* ≤ 0.05)*.

### Characterization of *CBF/DREB* Transcription Factors in Table Grapes

As a first step to characterize *CBF/DREBs* transcription factors, which belong to A1 subgroup, and because up to five different nomenclatures have been attributed (**Table 2**) for the different *CBF/DREBs* previously identified in *V. vinifera* (Xiao et al., 2006, 2008; Zhuang et al., 2009; Licausi et al., 2010; Cramer et al., 2014; Zhao et al., 2014; Carlow et al., 2017), we used the coding sequences of all of them to conduct a search in the 12X grape reference genome, V2.1 gene prediction (Vitulo et al., 2014). Six non-redundant *CBF/DREBs* transcription factors (*VIT_216s0100g00380*, *VIT_202s0025g04460*, *VIT_211s0016g02140*, *VIT_206s0061g01440*, *VIT_206s0061g01390*, *VIT_208s0007g03790*) were identified (**Table 2**). It is important to note that *CBF1* (Xiao et al., 2006) was not found in the 12X grape genome database. According to the nomenclature system developed by the International Grape Genome Program (IGGP) Supernomenclature committee (Grimplet et al., 2014), we decided to rename the different *CBF/DREBs* in the same way as the one proposed by Zhuang et al. (2009), since it refers to the family (DREB) and subgroup (A1) (**Table 2**). Taking into account that *CBF2* (*VviDREBA1-7* in this work), *CBF3* (*VviDREBA1*-6) and *CBF4* (*VviDREBA1-1*) have been extensively studied in grapevine cuttings exposed to low temperature, and that *CBF4* expression was modulated by high CO_2_ levels in Cardinal table grapes (Fernandez-Caballero et al., 2012), we have isolated them from table grapes cv. Autumn Royal using RT-PCR. VviDREBA1-1 consisted of 218 aa and shared 100% identity with VIT_216s0100g00380 and with VvCBF4 isolated from *V. vinifera* cv. Chardonnay (Xiao et al., 2008). VviDREBA1-6 (with 239 amino acids) was 83.3% identical with VIT_206s0061g01400 and the differences observed between them consisted of 10 variations of single amino acids and a protein fragment deletion of 28 amino acids in VIT_206s0061g01400. The percentage of identity of VviDREBA1-6 with VvCBF3 from *V. vinifera* cv. Chardonnay (Xiao et al., 2006) was 95.3, with differences in twelve amino acids. VviDREBA1-7 (253 amino acids) shared 99.6% identity with VIT_206s0061g01390, the difference being a variation of a single amino acid. Moreover, VviDREBA1-7 showed a 94.9% of identity with VvCBF2 (Xiao et al., 2006), presenting differences in 12 amino acids. It is important to point out that VviDREBA1-1 and VviDREBA1-6 presented an acidic predicted p*I* of 5.42 and 6.53, respectively, as occurs in dicot CBFs. However, VviDREBA1-7 showed a basic predicted p*I* of 9.71. Despite the p*I* difference, the putative activation domain at the C-terminus of the VviDREBA1-7 maintained a strongly acidic character with a p*I* of approximately 4.89, like the dicot CBFs.

**Table 2:** Summary of the *Vitis vinifera CBF/DREB* gene names designated in this study and in previous works.

Xiao et al., 2006, 2008	Zhuang et al., 2009	Licausi et al., 2010	Zhao et al., 2014	Cramer et al., 2014	Carlow et al., 2017	GrapegenDB 12Xv2 unique ID	Gene name in this study
VvCBF4	VvDREB-A1-1	VvERF022	VvDREB23	VviCBF1		VIT_216s0100g00380	VviDREBA1-1
VvCBF3	VvDREB-A1-2 VvDREB-A1-3 VvDREB-A1-4 VvDREB-A1-5 VvDREB-A1-6	VvERF021 VvERF023 VvERF024 VvERF025 VvERF026	VvDREB15 VvDREB07	VviDDF2 VviERF036 VviERF126	VvCBF6 VvCBF5	VIT_202s0025g04460 VIT_202s0025g04460 VIT_202s0025g04460 VIT_211s0016g02140 VIT_206s0061g01400	VviDREBA1-6
VvCBF2	VvDREB-A1-7	VvERF027	VvDREB06 VvDREB09	VviERF125 VviERF128	VvCBF8	VIT_206s0061g01390 VIT_208s0007g03790	VviDREBA1-7

**FIGURE 1:**
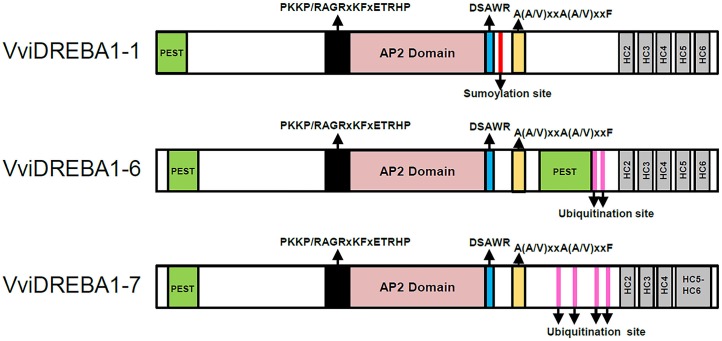
Schematic diagram of the three VviDREBA1s from *Vitis vinifera* cv. Autumn Royal showing conserved domains. The PEST sequences (green), the PKKPAGR motif (black), the AP2 domain (pink), the DSAWRL motif (blue), and the A(A/V)xxA(A/V)xxF motif (orange) are present in the three transcription factors. The hydrophobic clusters (HC2-HC6) (gray) which were present in the carboxy terminal region are indicated. A sumoylation target site (red line) was present in VviDREBA1-1 and ubiquitination sites (fuchsia lines) were present in VviDREBA1-6 and VviDREBA1-7.

Multiple sequence analysis showed that all deduced proteins exhibited the typical features of DREBA1 proteins, including an AP2 domain with the YRG, WLG, and RAHD motifs (Supplementary Figure S1). Moreover, these VviDREBA1 proteins also had the conserved valine (V14) in the 14th position in the AP2 domain. However, the conserved glutamic acid (E19) in the 19th position was replaced in VviDREBA1-6 by aspartic acid (D), also negative charged. The AP2 domain displayed a higher degree of amino acid identity than the N-termini and C-termini of the VviDREBA1 proteins. PEST motifs were predicted in the three VviDREBA1s in the N-terminal end close to the AP2 domain (**Figure 1**). At the C-terminal, a relevant PEST motif (more than 20 residues) was also predicted for VviDREBA1-6.

The DREBA1 signature sequences PKKP/RAGRxKFxETRHP (abbreviated PKKPAGR) and DSAWR, located immediately upstream and downstream of the AP2 domain, respectively (Jaglo et al., 2001), were also observed in the VviDREBA1 proteins (**Figure 1** and Supplementary Figure S1). However, only VviDREBA1-1 contained the conserved PKKPAGR consensus. Likewise, the domain A(A/V)xxA(A/V)xxF, with the underlined residues conserved in all known DREBA1 homologs (Xiong and Fei, 2006), was located in the downstream of the DSAWR motif in the three VviDREBA1s. However, the C-terminal LWSY motif (Dubouzet et al., 2003) was only conserved in the VviDREBA1-1 protein.

The hydrophobic cluster analysis of the C-terminus from VviDREBA1-1 and VviDREBA1-6 showed five hydrophobic clusters (HC2-HC6) (Supplementary Figure S2), which are known to be responsible for conferring *trans*-activation (Wang et al., 2005). By contrast, the analysis of VviDREBA1-7 revealed that HC5 and HC6 form a ‘mosaic cluster,’ which contained regular alternations of hydrophobic and non-hydrophobic residues, indicated as connecting lines.

The post-translational modifications were also evaluated through the prediction of ubiquitination and sumoylation sites associated with protein degradation and stability, respectively. A unique sumoylation site was predicted in VviDREBA1-1. However, two and four ubiquitination sites were predicted in VviDREBA1-6 and VviDREBA1-7, respectively.

### Expression Profile of *VviDREBA1s* Transcription Factors in Different Tissues of Autumn Royal Bunches Exposed to Low Temperature and High CO_2_ Levels

In a previous work, we denoted that the application of CO_2_ levels at 0°C in table grapes cv. Cardinal for 3 days induced the transcript accumulation of *VvcCBF1* in the pulp, and *VvcCBF4* (*VviDREBA1-1* in this work) in the pulp and rachis (Fernandez-Caballero et al., 2012). To study whether this effect is cultivar dependent and could be extensible to other *VviDREBA1s*, we analyzed the transcript accumulation of *VviDREBA1-1*, *VviDREBA1-6*, and *VviDREBA1-7* in different tissues of cv. Autumn Royal bunches through RT-qPCR.

**FIGURE 2:**
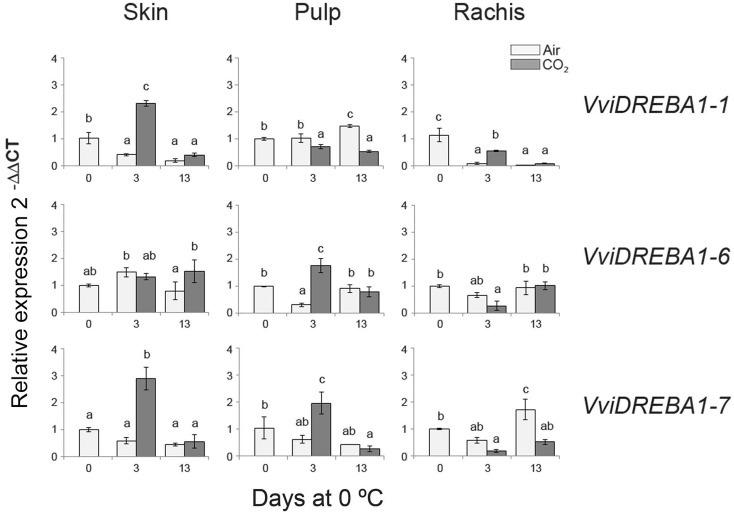
Expression pattern of *VviDREBA1s* in the skin, pulp and rachis of non-treated and CO_2_-treated bunches stored at 0°C up to 13 days. The relative quantification of transcripts was normalized to *Actin1* and the results were calculated relative to a calibrator sample (day 0) using the formula 2^-ΔΔCt^. Values are the mean ± SD, *n* = 6. The different letters on the bars indicate that means are statistically different using Duncan’s test (*P* ≤ 0.05).

The storage at low temperature of table grapes cv. Autumn Royal induced different changes in *VviDREBA1s* expression according to the tissue analyzed and the atmosphere composition (**Figure 2**). Thus, in the case of the skin, only the application of a 3-day gaseous treatment at 0°C significantly induced the expression of *VviDREBA1-1* and *VviDREBA1-7*, decreasing when table grapes were transferred to a normal atmosphere at 0°C. However, a slight but not significant accumulation of *VviDREBA1-6* transcript was observed in this tissue at 0°C regardless of the atmosphere composition. In the case of the pulp, whereas *VviDREBA1-6* and *VviDREBA1-7* transcript levels were induced by the application of a 3-day gaseous treatment, in the case of *VviDREBA1-1* an increase in the transcript accumulation was only observed after 13 days of storage of non-treated table grapes at 0°C. In the rachis, unlike in the skin and pulp, our results indicated that the gaseous treatment did not induce the expression of any *VviDREBA1s*, and only a significant accumulation of *VviDREBA1-7* was observed after 13 days at 0°C in non-treated bunches. By contrast, a decrease in *VviDREBA1-1* transcript levels was observed during the storage at 0°C regardless of the atmosphere composition, whereas *VviDREBA1-6* expression only decreased significantly in 3-day CO_2_ treated samples.

### Modulation of *VviDHNs* Expression in Different Tissues of Autumn Royal Bunches by Low Temperature and High CO_2_ Levels

**FIGURE 3:**
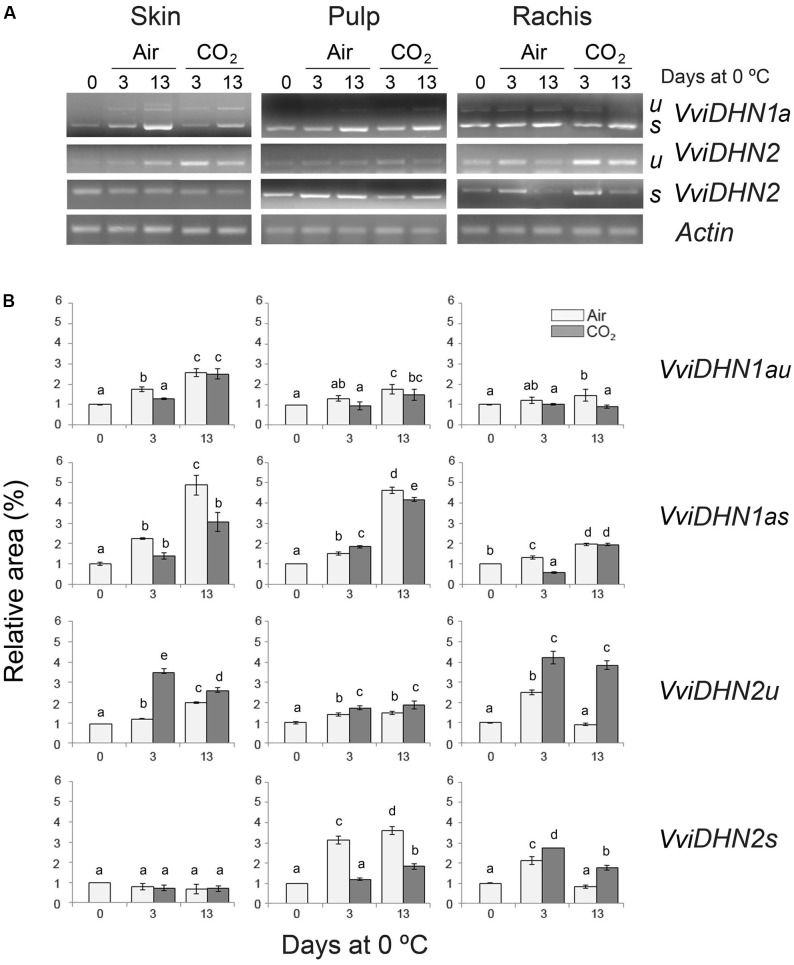
*VviDHN1a* and *VviDHN2* gene expression in the skin, pulp and rachis of *V. vinifera* cv. Autumn Royal bunches after a 3-day high CO_2_ treatment and low temperature storage. **(A)** The levels of *VviDHNs* and *Actin1a* (control) transcripts were determined by semi-quantitative RT-PCR analysis. u: fragments produced in unspliced transcripts; s: fragments produced in spliced transcripts. **(B)** The results were calculated by densitometry quantification of the band intensity of each sample using Adobe Photoshop and were expressed as a relative fold-change with respect to bunches before storage (day 0; relative area). Values are the mean ± SD, *n* = 3. The different letters on the bars indicate that means are statistically different using Duncan’s test (*P* ≤ 0.05).

**FIGURE 4:**
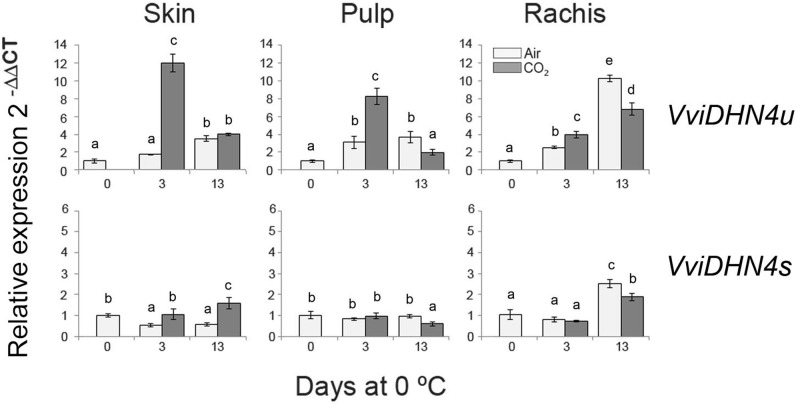
Relative gene expression of *VviDHN4* spliced and unspliced (*VviDHN4s* and *VviDHN4u*) in the skin, pulp and rachis of *V. vinifera* cv. Autumn Royal bunches after a 3-day gaseous treatment and low temperature storage. The relative quantification of transcripts was normalized with *Actin1* and the results were calculated relative to a calibrator sample (day 0) using the formula 2^-ΔΔCt^. Values are the mean ± SD, *n* = 6. The different letters on the bars indicate that means are statistically different using Duncan’s test (*P* ≤ 0.05).

To analyze the putative role of VviDREBA1s in the regulation of *DHNs*, we first studied the changes of spliced and unspliced *VviDHN1a*, *VviDHN2*, and *VviDHN4* mRNAs in fruit and non-fruit tissues of table grapes which were CO_2_-treated and non-treated and stored at 0°C. To this end, we performed semi-quantitative RT-PCR (*VviDHN1a* and *VviDHN2*) or RT-qPCR (*VviDHN4*). We observed that both *VviDHN1a* spliced and unspliced transcript levels were induced by storage at 0°C regardless of the atmosphere composition, although spliced transcripts were predominant in all the tissues analyzed (**Figure 3**). By contrast, differences between the spliced and unspliced transcript accumulation were observed in the case of *VviDHN2* and *VviDHN4* (**Figures 3**, **4**). Thus, although *VviDHN2* and *VviDHN4* unspliced forms were activated by exposure to 0°C both in treated and non-treated samples in all the tissues analyzed, the accumulation observed by applying the gaseous treatment after 3 days was significantly higher. However, *VviDHN2* spliced transcripts showed a tissue dependent regulation, remaining unchanged in the skin and increasing mainly by low temperature in the pulp of non-treated berries, whereas in the case of the rachis, higher levels of the spliced transcripts were reached in CO_2_-treated samples (**Figure 3**). By contrast, *VviDHN4* spliced transcript accumulation remained constant in the skin and pulp of treated and non-treated berries until day 13 where the levels increased in the skin of CO_2_ treated fruit, decreasing in the pulp. In the rachis, an increase was observed after 13 days at 0°C regardless of a gaseous pretreatment, with it being significantly higher in non-treated samples (**Figure 4**).

### Identification of *Cis*-Regulatory Elements in the Promoter Regions of *VviDHN2* and *VviDHN4*

**FIGURE 5:**
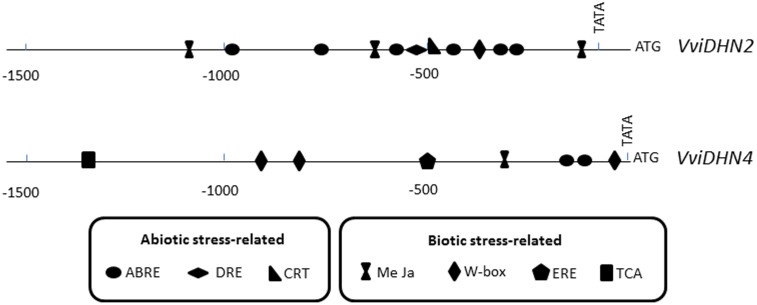
Schematic representation of biotic- and abiotic-related putative *cis*-acting regulatory elements in promoter regions of *VviDHN2* and *VviDHN4* genes.

The identification and *in silico* analysis of 1500 bp of *VviDHN2* and *VviDHN4* promoter regions from *V. vinifera* using the Genomatix suite of programs led to the identification of several putative *cis*-acting regulatory elements associated with abiotic and biotic stress responses (**Figure 5**). The abiotic stress-related elements included ABA-responsive elements (ABRE), dehydration-responsive elements (DRE), and C-repeat elements (CRT). Identified within the biotic stress-related elements were methyl jasmonate-responsive elements (MeJa), ethylene-responsive elements (ERE), salicylic acid-responsive elements (TCA), and elicitor-responsive elements (W-box). *VviDHN2* promoter harbored twelve *cis*-regulatory elements: eight related to abiotic stress and three related to biotic stress. Meanwhile, the *VviDHN4* promoter presented ten *cis*-regulatory elements: two related to abiotic stress and seven related to biotic stress. Moreover, it is important to point out that *VviDHN2* presented one DRE (ACCGAC core sequence) and one CRT (GCCGAC) motif, while *VviDHN4* did not present any of them.

Additionally, the promoter of *VviDHN1a* from table grapes cv. Cardinal was previously isolated (Rosales et al., 2014), and shared 98.4% identity with the corresponding sequence in the PN40024 grapevine reference genome. Among the ten *cis*-regulatory elements observed, a DRE motif was present.

### VviDREBA1-1 Is a CRT Binding Protein

**FIGURE 6:**
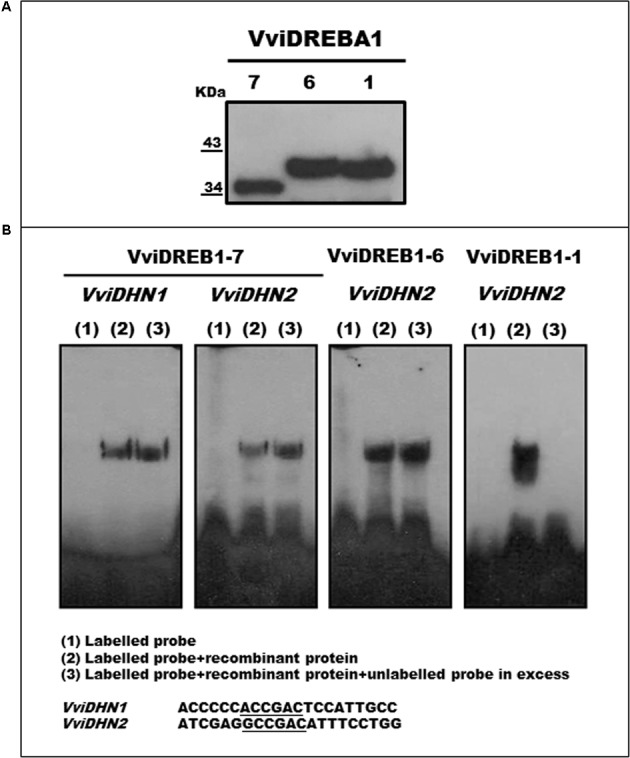
**(A)** Western blot of recombinant VviDREBA1s. **(B)** EMSA analyses were performed with recombinant VviDREBA1s proteins. DRE or CRT probes were labeled in the 3′end with biotin. For the competition assays, five hundred times of the unlabelled probe was added before the labeled probes. Sequences of the oligonucleotides containing the DRE or CRT motifs (underlined) used as probes are shown.

Purified VviDREBA1s recombinant proteins were obtained by Ni-NTA agarose and confirmed and visualized by Western Blot using the anti-6xHis monoclonal antibody (**Figure 6A**). EMSA analysis was performed to assess the capacity of the three VviDREBA1s to bind the DRE or CRT motifs. Only the probes from promoters of *VviDHN1a* and *VviDHN2* were used since they contained the target motifs (DRE or CRT). However, mutated versions of the mentioned probes were included. Our results showed that only VviDREBA1-1 was able to bind specifically to the *VviDHN2* CRT motif. In this case, the binding activity was abolished by competition with large amounts of unlabeled probe (**Figure 6B**). Likewise, we have observed that the change in the flanking regions of the CRT motif did not abolish the binding activity although the intensity was reduced (Supplementary Figure S3). By contrast, no binding or unspecific binding was observed in the assays performed with the VviDREBA1-1 and the probes harboring DRE motif; or with VviDREBA1-6 and VviDREBA1-7 proteins containing the DRE or CRT motifs; or with the mutated probes (data not shown). It is important to remark that only the results which show specific or unspecific binding have been included in **Figure 6B**.

## Discussion

Mature table grapes are non-climacteric fruit with a relatively low respiration rate, but are subject to serious water loss, softening and fungal attack after harvest. Grapes are classified as a non-chilling sensitive fruit and postharvest storage around 0°C is recommended to maintain their quality. However, we have observed that table grapes cv. Cardinal are sensitive to temperature shifts from the field to storage at 0°C with increasing rachis browning, water loss and ion leakage, as well as the activation of phenylpropanoid and pathogenesis related gene expression, whereas a 3-day high CO_2_ pretreatment at 0°C avoids and/or modifies these changes (Sanchez-Ballesta et al., 2006; Rosales et al., 2016). In the present study, we have observed that the application of high CO_2_ levels for 3 days at 0°C was effective in maintaining the quality of table grapes other than Cardinal, such as Autumn Royal. Previous results in Cardinal berries showed that the CO_2_ treatment significantly affected the moisture content of all tissues of the bunch, except for pulp tissues (Goñi et al., 2011). However, similar values of moisture content were quantified in 3-day CO_2_-treated and non-treated Autumn Royal berries stored at 0°C, as was observed in strawberries (Blanch et al., 2012). On the other hand, CO_2_-treated table grapes showed a low weight loss percentage in comparison to non-treated bunches during storage at 0°C. This effect has been also reported by Martínez-Romero et al. (2003) in table grapes, and in other types of fruit such as nectarines (Retamales et al., 2000), cucumbers (Wang and Qi, 1997), and cherries (Kappel et al., 2002) stored in modified atmosphere packaging at low temperature. Concerning the rachis browning index, it was lower in Autumn Royal bunches treated with CO_2_ for 3 days than in non-treated bunches, as has been observed in Cardinal table grapes (Rosales et al., 2013). However, this is not a common response to high CO_2_ levels. Thus, rachis appearance was adversely affected in Thompson Seedless and Red Globe bunches stored up to 40 or 45 days at low temperature under controlled atmosphere CO_2_ levels ≥15 kPa, respectively, and after 4 days of shelf-life (Retamales et al., 2003). The beneficial effect of the gaseous treatment on the rachis appearance of Autumn Royal bunches could be explained by lower water loss from the rachis under this treatment (Sanchez-Ballesta et al., 2006). Accordingly, in Cardinal bunches the relative water content of the rachis, an indicator of water balance status, decreased considerably throughout storage at 0°C in non-treated bunches whereas the application of 3-day high CO_2_ levels reduced this effect (Rosales et al., 2013).

In recent studies, we have shown that maintaining Cardinal table grape quality by the application of a 3-day CO_2_ treatment seems to be an active process which requires the activation of transcription factors, such as *ERFs*, belonging to the AP2/ERF transcription factor family (Romero et al., 2016; Rosales et al., 2016). In addition, we have observed that the gaseous treatment applied to Cardinal bunches induced the expression of other members of this transcription factor family such as *CBF/DREB* (Fernandez-Caballero et al., 2012). The CBF/DREB proteins play a crucial role in abiotic stress-mediated gene expression, representing one of the most attractive regulons for breeding programs (Zandkarimi et al., 2015) to cope with cold stress. Different studies have indeed shown the relationship between *CBF/DREB* expression and cold tolerance in different fruit such as tomato, peach, kiwifruit, and mango during low temperature storage in combination with different postharvest treatments (Liang et al., 2013; Ma et al., 2014; Zhang et al., 2017). However, with the exception of the study mentioned above, the relationship of these *CBF/DREBs* with the different sensitivity to temperature shifts induced in fruit by high CO_2_ levels is still unknown. Thus, we have isolated three *DREBA1s* from Autumn Royal table grapes and characterized their expression pattern in the skin, pulp and rachis during postharvest storage at low temperature with a 3-day high CO_2_ treatment. In this work, we observed that gene expression varied according to the *VviDREBA1* analyzed and the tissue studied. The storage of Autumn Royal bunches at low temperature under normal atmosphere was enough to activate the expression of *VviDREBA1-1* in the pulp and *VviDREBA1-7* in the rachis. Likewise, as was observed in Cardinal grapes (Fernandez-Caballero et al., 2012), the 3-day high CO_2_ treatment at 0°C modulated the expression of *VviDREBA1s* by activating *VviDREBA1-1* and *VviDREBA1-7* in the skin, and *VviDREBA1-6* and *VviDREBA1-7* in the pulp. On the other hand, it is important to note that the effect of low temperature storage under normal atmosphere modulated *VviDREBA1-1* and *VviDREBA1-7* after 13 days of storage, while the three *VviDREBA1s* were induced at the end of the 3-day gaseous treatment at 0°C. This temporal difference could be important to help table grapes face temperature shifts at 0°C. Thus, Santiam tomato fruit showed less sensitivity to chilling than Lichum tomatoes, reflected in the lowest chilling injury index, ion leakage and malondialdehyde content. Meanwhile, *LeCBF1* transcript accumulation was higher in Santiam tomatoes, indicating that there was a swift genetic response to chilling stress, which was positively correlated with cold tolerance of the cultivar (Zhao et al., 2009).

It is known that the activation of CBF/DREBs improves the expression of downstream target genes, especially those encoding LEA proteins, including *DHNs*, as these transcriptional activators can bind to the *cis* DRE/CRT motif present in the promoter regions of these genes. Overexpression of *AtCBF1*–*AtCBF3* in transgenic Arabidopsis plants resulted in an increase in freeze tolerance and in the activation of different *COR* genes, including *DHNs*, at room temperature (Gilmour et al., 2004). A comparative study of two citrus species (*Poncirus* and *Citrus*) with different levels of freezing tolerance, showed a correlation between *CBF1* expression and the degree of tolerance observed (Champ et al., 2007). These authors further demonstrated that *CBF1* specifically recognized the consensus sequence (CCGAC) of the DRE/CRT elements from the dehydrin promoter of *Poncirus trifoliata*. To study the role of VviDREBA1s in the regulation of *VviDHNs*, we first studied the gene expression of spliced and unspliced *VviDHN1*, *VviDHN2*, and *VviDHN4* mRNAs in different tissues of Autumn Royal bunches, both CO_2_-treated and non-treated with storage at 0°C. *VviDHN1a, VvDHN2*, and *VviDHN4* are known to undergo alternative splicing (Fernandez-Caballero et al., 2012; Yang et al., 2012). Intron retention during *VviDHNs* pre-mRNA processing leads to the production of mRNAs with premature stop codons in the intron that give rise to truncated proteins without the K segment, which is characteristic of this protein family. Likewise, Lee et al. (2016) reported that *OsCYP19-4* (the primary cyclophilin 19-4 transcript from rice) was able to generate up to eight alternative splice forms via two types of regulated splicing events, intron retention and exon skipping, under low temperatures. Thus, in the case of Arabidopsis, temperature stress (cold and heat) dramatically altered the alternative spliced of pre-mRNAs of several serine/arginine-rich proteins, a conserved family of splicing regulators in eukaryotes (Palusa et al., 2007). Although the functionality of unspliced DHNs is not well known, in a previous work we demonstrated that the unspliced DHN1a variant from *V. vinifera* cv. Cardinal slightly interacted with DNA (Rosales et al., 2014). In this work, we have observed that unspliced forms showed a higher regulation degree than spliced forms after the CO_2_ treatment. Therefore, with the exception of unspliced *VviDHN1a* and *VviDHN4* in rachis, the 3-day CO_2_ treatment regulated the transcript levels in the unspliced forms in the different tissues of Autumn Royal.

The study of the VviDHNs promoter regions showed that, whereas the *VviDHN2* promoter region showed the presence of one DRE element and one CRT element, *VviDHN4* did not show any of them. Likewise, previous results showed the presence of a DRE element in the VviDHN1 promoter (Rosales et al., 2014). Through EMSA assays, our work provides evidence that the recombinant protein VviDREBA1-1 was the only one which showed specific binding to the CRT element (GCCGAC) presented in the VviDHN2 promoter, while no binding was observed to the DRE element (ACCGAC). In a recent work (Carlow et al., 2017), transient transactivation assays showed that all *V. riparia* CBFs except CBF5 activate via a CRT or DRE promoter element, whereby *V. riparia* CBF3 (homolog to Vv1DEBA1-6) and CBF4 (homolog to Vv1DEBA1-1) prefer a CRT element. It is known that not all CBF/DREBs have the same affinity and specificity for DRE/CRT elements. Thus, BNCBF17 from *Brassica napus* showed lower binding specificity than BNCBF5 to the core CCGAC sequence when this was mutated (Gao et al., 2002), while HvCBF1 from barley showed preference for binding to the sequence TTGCCGACAT, which contained the CRT motif instead of DRE (Xue, 2002). Likewise, the analysis of the promoters of COR genes induced in Arabidopsis plants which overexpressed CBFs demonstrated that variations in the sequences around the CRT element could affect the activation of the promoters (Maruyama et al., 2004). In this regard, our results showed that the region flanking the CRT element must have a role in the affinity of the VviDREBA1s, since changes in some nucleotides did not abolish the binding observed with VviDREBA1-1, but its intensity was reduced. Although more studies are needed to be able to understand the binding specificity of CBF/DREBs for target sequences, the results are in concordance with previous results reported by Nassuth et al. (2014) which suggest that CBF/DREBs paralogs in a plant, and possibly orthologs from different species, have a unique preference for DRE/CRT sequences. At this point it is important to remark that the three VviDREBA1s contained the signature sequences, PKKPAGR and DSAWR, flanking the AP2 domain which distinguishes this subgroup of transcription factors from the other AP2/ERF family members (Jaglo et al., 2001). Canella et al. (2010) showed that deletions or mutation in the PKKPAGR sequence greatly impaired the ability of AtCBF1 to induce expression of target *COR* genes because of its inability to bind to the DRE/CRT element indicating that amino acids beyond the AP2 domain are required. In the specific case of DREBA1s from Autumn Royal, we observed that whereas the PKKPAGR motif was well conserved in VviDREBA1-1 (PKKRAGRxKFxETRHP), there were three (HKRKAGRxKFxETRHP) and five (HKRKTGRxKFxKTRHP) amino acids which were different in VviDREBA1-6 and VviDREBA1-7, respectively. In *Vitis*, Xiao et al. (2006) observed that despite the three amino acid changes detected in CBF1, targeting and transactivation experiments denoted that CBF1 still functions. Nevertheless, transient activation experiments suggested that *V. riparia* CBF4 was a more effective activator than VrCBF1 (Xiao et al., 2008), and that Arabidopsis transgenic plants which overexpressed *V. riparia* CBF1 and CBF4 presented higher amounts of *COR* genes in the CBF4 lines (Siddiqua and Nassuth, 2011). Likewise, Nassuth et al. (2014) corroborated previous results and showed, with a new system of transactivation, that *VrCBF1* and *VrCBF4* transcription factors probably activate different overlapping sets of genes, and therefore play unique roles in cold acclimation. Thus, this evidence could be related to the changes observed in the PKKPAGR motif of *Vitis* CBFs.

**FIGURE 7:**
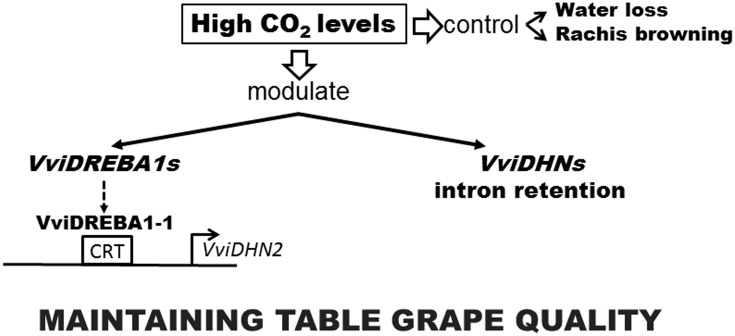
A scheme depicting events of Autumn Royal table grapes in response to high CO_2_ levels during postharvest storage at 0°C.

Overall, our results demonstrated that the 3-day gaseous treatment was effective in maintaining table grape quality regardless of the cultivar and modulated the expression of *VviDREBA1s* transcription factors in a tissue dependent way. Furthermore, the application of high levels of CO_2_ regulated the retention of introns in the transcripts of the dehydrins in a different manner and in all the tissues analyzed. On the other hand, VviDREBA1-1 was the only transcription factor analyzed that presented *in vitro* binding capacity to the CRT element of the VviDHN2 promoter region, indicating that the transcriptional regulation of *VviDHN1a* and *VviDHN4* would be carried out by activating other independent routes of these transcription factors (**Figure 7**). These results, together with the fact that *VviDREBA1-1* gene expression was induced by high CO_2_ levels in the skin of Autumn Royal fruit and in the pulp and rachis of Cardinal bunches (Fernandez-Caballero et al., 2012), make this transcription factor a good candidate for further research with the aim of improving table grape quality.

## Author Contributions

MV-H and IR contributed equally to this work. MV-H: Participated in the table grape quality assessments, carried out the RNA extraction, RT-qPCR analysis, semiquantitative RT-PCR, statistical analyses. IR: Carried out the VviDREBA1s isolation and the production of recombinant proteins, the EMSA analysis, edited and collaborated in the first draft of the manuscript. ME: Conceived the postharvest table grapes storage experience, participated in the table grape quality assessments and critically revised the manuscript. CM: Participated in the table grape quality assessments and critically revised the manuscript. MS-B: conceived the VviDREBA1s and DHNs study, supervised and coordinated the experiments, interpreted the results and prepared the first draft of the manuscript. All authors have read and approved this manuscript.

## Conflict of Interest Statement

The authors declare that the research was conducted in the absence of any commercial or financial relationships that could be construed as a potential conflict of interest.

## Footnotes

1 http://genomes.cribi.unipd.it/grape/

2 http://www.ch.embnet.org/software/LALIGN_form.html

3 http://www.ubpred.org/

4 http://sumosp.biocuckoo.org/online.php

5 http://emboss.bioinformatics.nl/cgi-bin/emboss/epestfind

6 http://www.genomatix.de
