# Supplementary material for: Deciphering the Role of CBF/DREB Transcription Factors and Dehydrins in Maintaining the Quality of Table Grapes cv. Autumn Royal Treated with High CO2 Levels and Stored at 0°C
Source: Front Plant Sci. 2017 Sep 20;8:1591. doi: 10.3389/fpls.2017.01591 (PMC5609105; doi:10.3389/fpls.2017.01591)

Supplementary Figure S1. Alignment of the deduced amino acid sequences of VviDREBA1-1 (MF445007), VviDREBA1-6 (MF445008) and VviDREBA1-7 (MF445009) from *V. vinifera* cv. Autumn Royal; VvCBF2 (AY390376), VvCBF3 (AY390375) and VvCBF4 (DQ497624) from *V. vinifera* cv. Chardonnay and VIT_216s0100g00380, VIT_206s0061g01390, VIT_206s0061g01400 from 12X grape reference genome. Identical amino acids and conservative substitutions are shown on a black or gray background, respectively. The dashes indicate gaps introduced to better alignment. The PKKPAGR, AP2, DSAWR, A(A/V)xxA(A/V)xxF and LWSY domains are indicated with different colors. The hydrophobic clusters (HC2-HC5) are boxed.

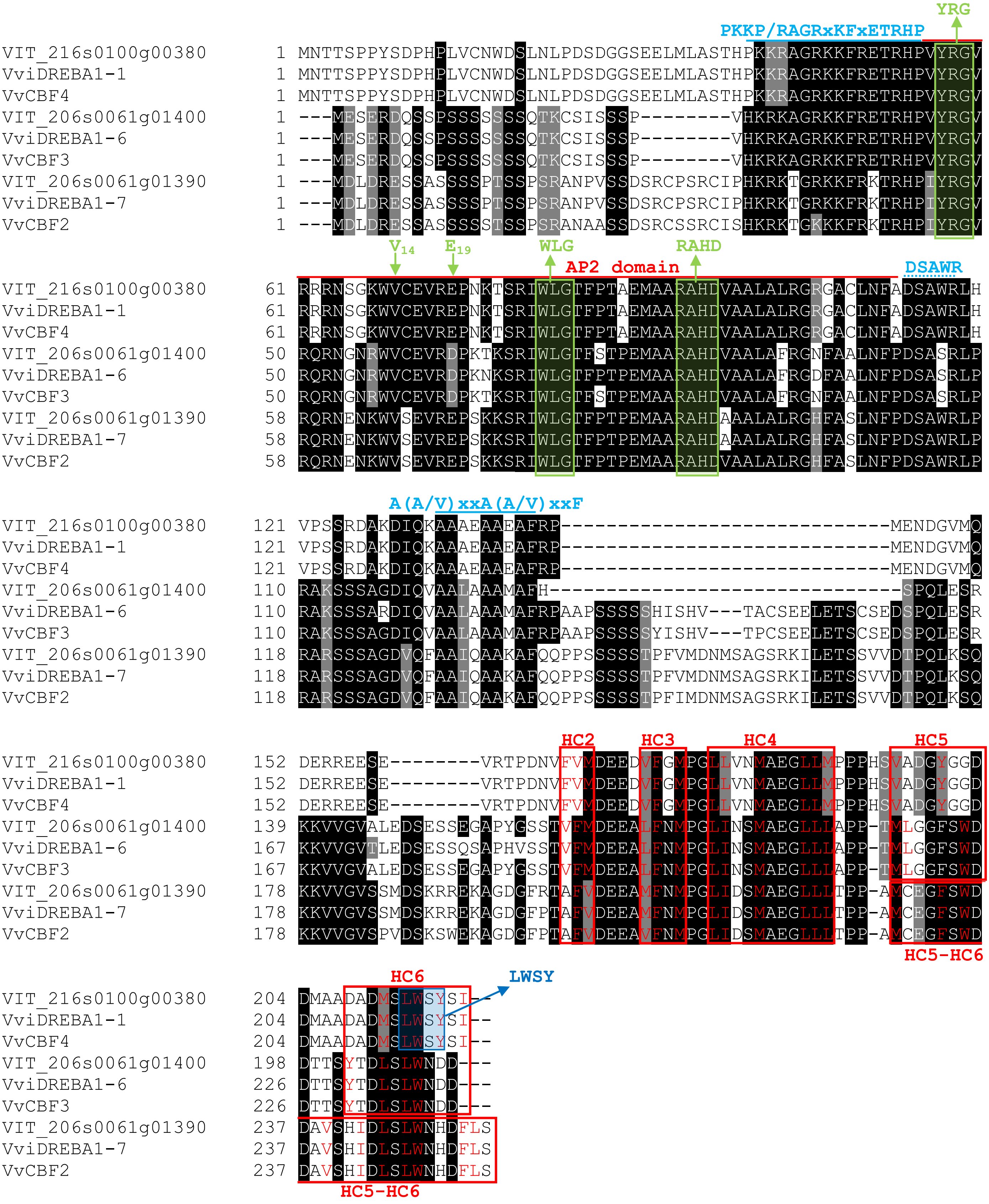

Supplement: Supplementary file 2 [file Image_1.PDF]
