# Supplementary material for: Deciphering the Role of CBF/DREB Transcription Factors and Dehydrins in Maintaining the Quality of Table Grapes cv. Autumn Royal Treated with High CO2 Levels and Stored at 0°C
Source: Front Plant Sci. 2017 Sep 20;8:1591. doi: 10.3389/fpls.2017.01591 (PMC5609105; doi:10.3389/fpls.2017.01591)

Supplementary Figure S2. Hydrophobic cluster analysis (HCA) of AtCBF1 (AY667247), VvDREBA1-1, VvDREBA1-6, VvDREBA1-7 COOH-terminal 60 amino acid residues. Each hydrophobic cluster is identified above the HCA output. Classic symbols were used for amino acids except for proline (★), glycine (♦), theronine (☐) and serine (▣).

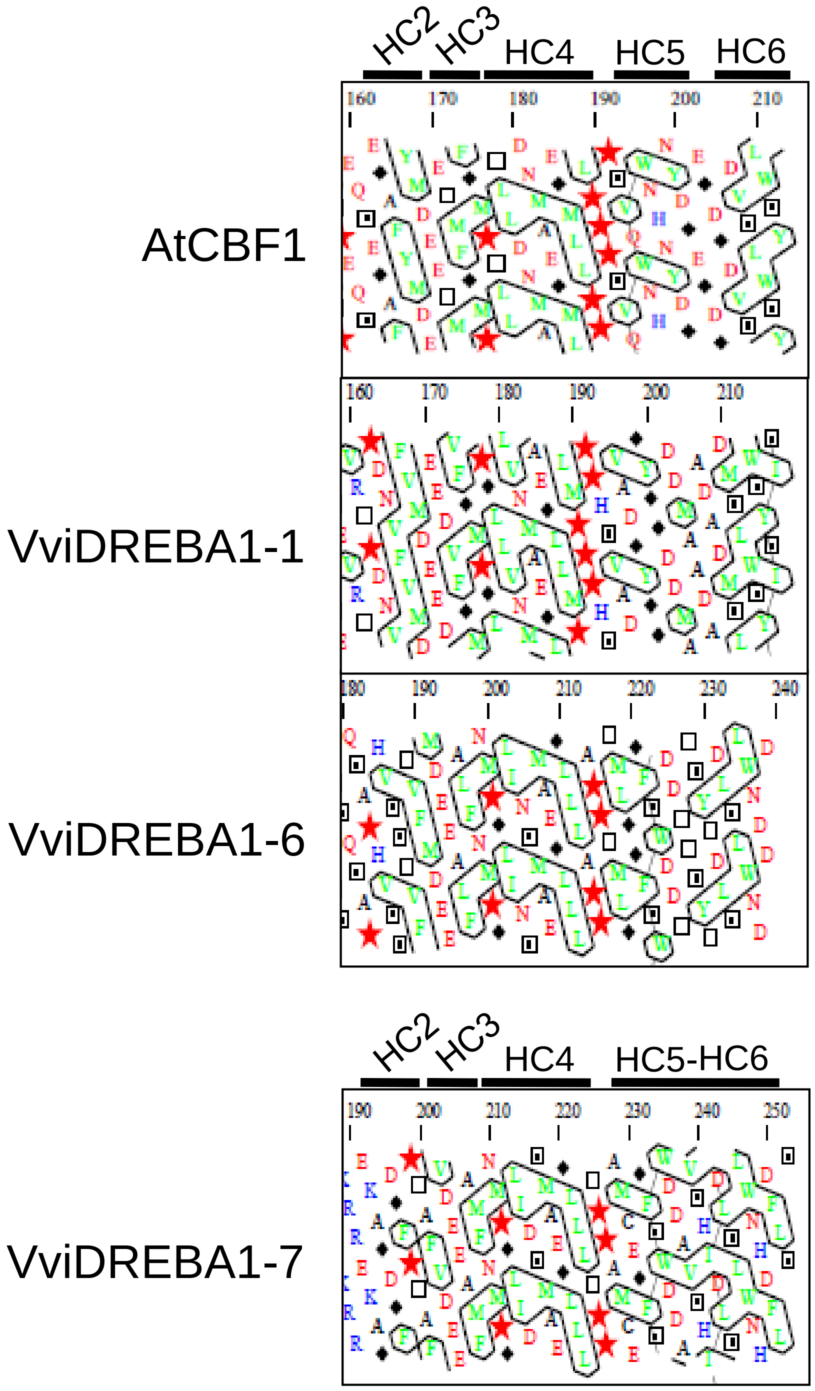

Supplement: Supplementary file 3 [file Image_2.PDF]
