# Supplementary material for: Deciphering the Role of CBF/DREB Transcription Factors and Dehydrins in Maintaining the Quality of Table Grapes cv. Autumn Royal Treated with High CO2 Levels and Stored at 0°C
Source: Front Plant Sci. 2017 Sep 20;8:1591. doi: 10.3389/fpls.2017.01591 (PMC5609105; doi:10.3389/fpls.2017.01591)
Supplement: Supplementary file 4 [file Image_3.PDF]

Supplementary Figure S3. EMSA analysis were performed with recombinant VviDREBA1-1 protein and *VviDHN2* or *VviDHN2** probes containing the CRT core motif. *VviDHN2** was designed with different flanking core nucleotides. Probes were labelled in the 3´end with biotin. Five hundred times of the unlabeled probe was added prior to the labelled probe for the competition assay. *Cis*-elements used in the EMSA are shown and CRT core is underlined.

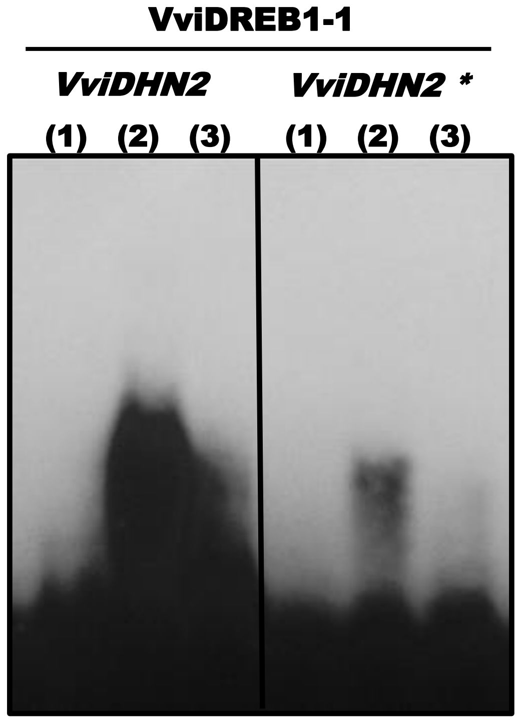

(1) Labelled probe
(2) Labelled probe+recombinant protein
(3) Labelled probe+recombinant protein+unlabelled probe in excess

VviDHN2 **ATCGAGGCCGACATTTCCTGG**
VviDHN2 * **TGCTTGCCGACGCCAATAGA**
